# Added value of video edutainment on android handsets in home visits to improve maternal and child health in Bauchi State, Nigeria: Secondary analysis from a cluster randomised controlled trial

**DOI:** 10.1177/20552076241228408

**Published:** 2024-02-13

**Authors:** Umaira Ansari, Khalid Omer, Amar Aziz, Yagana Gidado, Hadiza Mudi, Ibrahim Sabo Jamaare, Neil Andersson, Anne Cockcroft

**Affiliations:** 1 Centro de Investigación de Enfermedades Tropicales, 341132 Universidad Autónoma de Guerrero, Acapulco, Mexico; 2 Federation of Muslim Women's Association of Nigeria, Bauchi, Nigeria; 3 Bauchi State Primary Health Care Development Agency, Bauchi, Nigeria; 4 CIET-PRAM, Department of Family Medicine, 5620 McGill University, Montreal, Canada

**Keywords:** Video edutainment, android handsets, home visits, intervention research, maternal and child health, male involvement

## Abstract

**Objective:**

A trial of evidence-based health promotion home visits to pregnant women and their spouses in northern Nigeria found significant improvements in maternal and child health outcomes. This study tested the added value for these outcomes of including video edutainment in the visits.

**Methods:**

In total, 19,718 households in three randomly allocated intervention wards (administrative areas) received home visits including short videos on android handsets to spark discussion about local risk factors for maternal and child health; 16,751 households in three control wards received visits with only verbal discussion about risk factors. We compared outcomes between wards with and without videos in the visits, calculating the odds ratio (OR) and 95% confidence interval (95%CI) of differences, in bivariate and then multivariate analysis adjusting for socio-economic differences between the video and non-video wards.

**Results:**

Pregnant women from video wards were more likely than those from non-video wards to have discussed pregnancy and childbirth often with their husbands (OR 2.22, 95%CI 1.07–4.59). Male spouses in video wards were more likely to know to give more fluids and continued feeding to a child with diarrhoea (OR 1.61, 95%CI 1.21–2.13). For most outcomes there was no significant difference between video and non-video wards. The home visitors who shared videos considered they helped pregnant women and their spouses to appreciate the information about risk factors.

**Conclusion:**

The lack of added value of the videos in the context of a research study may reflect the intensive training of home visitors and the effective evidence-based discussions included in all the visits. Further research could rollout routine home visits with and without videos and test the impact of video edutainment added to home visits carried out in a routine service context.

## Introduction

Mass media entertainment education or edutainment can lead to modest shifts in health behaviour and outcomes.^1,2^ A recent review of 126 studies of entertainment education noted that 77 reported changes in intermediate outcomes (such as knowledge and attitudes) only, 23 reported sustained behaviour and social changes and 26 reported little or no effect.^3^ Video edutainment in community interventions in Africa has had promising results.^4,5^ A trial of edutainment in a community ‘film-festival’ in 112 villages in rural Uganda reported increased willingness to report violence against women in people exposed to the edutainment, and a reduction in the experience of violence.^6^ Video edutainment can also be used in interactions with individuals or households during home visits, when videos can be screened on handsets. A video edutainment intervention in homes among Mexican-origin families in the USA improved dietary choices among mothers and improved dietary intake.^7^ In Thailand, a before-after study of an edutainment module on mobile tablets reported improved knowledge and perceptions about childhood immunisation among mothers in hard-to-reach, under-vaccinated populations.^8^ These studies measured the impact of edutainment videos as part of an overall home visits package; they do not provide information about the specific contribution of the edutainment videos to the impact on the targeted outcomes.

Maternal mortality and morbidity remain very high in Nigeria^9^ and there is limited access to effective maternal care services.^10^ A trial of universal home visits (including all households in an area) to pregnant women and their husbands in Bauchi State, northern Nigeria, aimed to reduce maternal and early childhood morbidity. Female and male home visitors visited all pregnant women and their male spouses and discussed with them evidence about risk factors and maternal and child health, from surveys in Bauchi State.^11^ They shared the evidence either verbally alone or with added videos about the risk factors. The published protocol provides full details of the methods of the cluster randomised controlled trial.^12^ The home visits intervention significantly improved maternal health outcomes and the targeted risk factors, child health outcomes and male knowledge and attitudes about reproductive and child health.^13–15^ The objective of the study described here was to measure whether adding video edutainment to support the discussion about risk factors in the home visits produced any *additional* impact on the measured outcomes, over and above that of the visits themselves. We followed the Consolidated Standards Of Reporting Trials (CONSORT) guidelines in reporting the study.^16^

## Methods

### Setting

Bauchi state in north-eastern Nigeria has a mostly Muslim population of about 5 million. Toro local government authority (LGA) is the largest LGA in the state. The society is conservative, polygamy is common and family sizes are large. Socio-economic and health indicators are below the national average. In Bauchi, 73% of women have no formal education compared with 38% of women nationally.^17^

Maternal mortality in Nigeria is among the highest in the world,^9^ with the situation worse in Bauchi State.^18^ Child outcomes are poor in the State: only 19.6% of Bauchi children aged 12 to 23 months received all basic vaccinations compared with 31.3% of children nationally. The 15-day prevalence of diarrhoea is 34.1% among Bauchi children, compared with 12.8% nationally.^17^

Nigeria has a thriving local movie industry, popularly referred to as Nollywood. Locally made soap operas are very popular in Bauchi State. Previously in Bauchi, soap opera style video edutainment to share survey findings about maternal and child health was well received when screened in communities.^19^

### Trial design

The published protocol of the overall trial describes the stepped-wedge design.^12^ Three pairs of wards (administrative areas) in Toro LGA received the home visits, starting at yearly intervals. The overall trial compared outcomes in visited wards with those in not-yet-visited wards. To measure the impact of adding video edutainment in the home visits, the subject of this manuscript, we conducted a parallel group randomised controlled trial within the intervention arm of the overall trial. We allocated one of each pair of wards (total three wards) to receive home visits with videos, while the other ward in the pair (total three wards) received the home visits without the videos. We compared outcomes between wards including videos in the home visits (video wards) and wards without videos in the home visits (non-video wards).

Following a mixed methods approach, in addition to the quantitative measurement of added value of the video edutainment, we collected qualitative data about perceptions of the videos. We interviewed home visitors about their views of the utility of the videos and showed the videos to women and men in communities and asked about their understanding of the depicted scenes.

### Participants

All six wards receiving home visits in the main trial were eligible for this trial of the added value of videos in the visits. All households and all pregnant women and their male spouses were eligible to receive visits and all children born to mothers visited during the intervention period were eligible for inclusion once they reached 12 to 18 months of age. The study included 19,718 households visited in wards including videos in the visits and 16,751 households visited in wards without videos in the visits (see ‘Participant flow in the study’ below). The primary targets of the video edutainment videos were pregnant women and their male spouses, in those wards allocated to receive edutainment as part of the home visits.

### The intervention

The intervention took place between 2016 and 2019. Female home visitors visited all pregnant women every 2 months during the pregnancy, shortly after the birth and about 1 year later when the child was 12 to 18 months old.^12^ Male home visitors visited the male spouses of the pregnant women, also every 2 months during the pregnancy and after the birth. The home visitors presented and discussed (with or without videos) evidence from Bauchi State about four risk factors for maternal health: experience of domestic violence in pregnancy, heavy work during pregnancy, lack of basic knowledge of danger signs and lack of communication between spouses about pregnancy and childbirth.^11^ They also presented and discussed evidence about child health, including prevention and management of childhood diarrhoea and child immunisation. In the three non-video (control) wards, the visitors presented and discussed the evidence verbally only; they did not share videos. In the three video (intervention) wards, the home visitors presented and discussed the evidence verbally and also showed short videos about the risk factors. They shared the videos with the pregnant women and their male spouses by playing them on android handsets. They showed three videos about maternal health at each visit during the pregnancy and added two videos about child health in their visits late in pregnancy and immediately after the birth. Most of the visits with videos lasted about 30 minutes, and those with the additional videos about child health lasted about 45 minutes.

The five short videos, each about 4 minutes long, took the form of soap opera dramas popular in the area. They depicted people's attitudes and beliefs about the maternal and child health risk factors, illustrated the impacts of these risk factors and showed how actions by households could protect the health of pregnant women and small children. Each video concluded with a summary and advice from an actor in the role of a local community leader. A local production team filmed the videos in a rural community in Bauchi State. Local actors played the roles of characters in the dramas, speaking in the Hausa language. Research team members supported production of the videos during filming and editing. Government officers from the Ministry of Health, the State Primary Health Care Development Agency and Toro LGA viewed the first cut of the videos and made suggestions for creating the final versions. The videos are available with English subtitles at https://youtube.com/playlist?list=PL17QiK-Lpq1zdQJoZwkhFMKWJYiuVhMwl.

### Outcomes measured in the study

This trial compared outcomes among women, children and male spouses between video and non-video wards after one year of home visits in each pair of wards. Data collection relied on Open Data Kit (ODK) software.^20^ The home visitors administered electronic questionnaires to pregnant women and their spouses, entering their responses into the questionnaires on android handsets and sending them via the cellular network to a central server, from where the research team downloaded the dataset for analysis.

Measured maternal health outcomes included: knowledge of danger signs during pregnancy and childbirth (number of correct signs known), reduction in heavy work before the start of the third trimester, often discussing pregnancy and birth with the spouse and not experiencing verbal or physical domestic violence during pregnancy. They also included indicators of use of health services during pregnancy (antenatal care visits) and childbirth (facility or home) and of health complications in pregnancy (bleeding, pre-eclampsia) and childbirth (postnatal sepsis). Measured child health outcomes included maternal knowledge about prevention (hygiene) and management (increased fluids, continued feeding, avoidance of anti-diarrhoea medicines) of diarrhoea, 15-day prevalence of diarrhoea, management of last episode of diarrhoea and immunisation status of the child. Measured outcomes among male spouses included knowledge of danger signs during pregnancy and childbirth, views about heavy work during pregnancy, discussion of pregnancy and childbirth with the spouse and knowledge about prevention and management of childhood diarrhoea.

### Sample size

The sample size for this trial was fixed by the sample size for the overall trial of the impact of the home visits. The sample size for the overall trial was set to allow detection of a 20% reduction in pregnancy and childbirth complications (80% power at the 5% level, *k* = 0.05).^12^ Assuming the visits reduced post-partum infection by 20%, we estimated that a comparison of the three video wards with the three non-video wards would be able to detect a further improvement of 15% (80% power at 5% level, *k* = 0.05).^12^

### Randomisation and blinding

An epidemiologist not involved in the fieldwork (NA) used a random number sequence generator to randomly allocate one ward in each pair of wards to receive the home visits with videos. In each video ward, all the households had videos in the home visits, while in each non-video ward, none of the home visits included videos.

The research team knew which wards had videos with the home visits; they trained the home visitors to show the videos. In the video wards, participants and home visitors were of course aware of the videos. Other than the showing of the videos, all field protocols, procedures and data collection instruments were the same for video and non-video wards.

### Statistical methods

Analysis relied on CIETmap software,^21^ which provides a user interface with the R statistical language. We compared the frequency of socio-economic variables in the video and non-video groups of women (maternal outcomes), children aged 12 to 18 months (child health outcomes) and male spouses (male knowledge outcomes). Bivariate analysis examined the associations between exposure to videos during the home visits and maternal, child health and male knowledge outcomes. We report odds ratios and 95% confidence intervals (95%CIs), adjusted for clustering by ward.^22^ Multivariate analysis used the Mantel–Haenszel procedure^23^ with adjustment for clustering by ward. We created models including the video exposure variable and socio-economic variables relevant to each set of outcomes: maternal health, child health and male knowledge and attitudes. We report adjusted odds ratios and cluster adjusted 95%CIs for the associations between video exposure and the outcomes.

### Qualitative data collection and analysis

Towards the end of the trial period, members of the local research team interviewed female and male home visitors to ask about their experiences of showing videos during the visits. The semi-structured questionnaire asked the home visitors about their perception of the reaction of pregnant women and their male spouses when they watched the videos: whether they apparently understood the messages in the videos, how they interacted with the visitor while watching the videos and whether they thought the visited women and men understood the topics better than would have been possible without the videos. The interviewers took detailed notes during each interview, including some verbatim quotes. They interviewed six female and six male visitors from two urban, two rural and two remote communities. They did not directly interview visited women or their spouses.

The local research team also showed the videos without any additional explanation to six men and six women in two urban, two rural and two remote non-trial communities in Bauchi State and interviewed them afterwards, using a semi-structured guide, to assess how well they understood the content and main messages in the videos. They took detailed notes during each interview.

The lead author, with experience of qualitative and quantitative research in Bauchi, conducted an inductive thematic analysis^24^ of the interview reports to identify and group themes arising. She extracted key quotes illustrating different themes.

### Ethical considerations

Community leaders in the participating wards gave approval for the home visits in their communities and actively promoted the visits. Home visitors obtained oral informed consent from all respondents for every interview, at every visit. We treated all responses from participants as confidential. The Bauchi State Health Research Ethics Committee and the McGill Faculty of Medicine IRB approved the trial.

## Results

### Participant flow in the study

The trial took place in 100 catchment areas (each about 300–380 households): 58 in video wards and 42 in non-video wards. The home visits covered a total of 19,718 households in video wards and 16,751 households in non-video wards. For maternal outcomes, we analysed 3785 women completing pregnancies in video wards and 3899 in non-video wards. For child outcomes, we analysed 1090 children aged 12 to 18 months born to mothers in video wards and 706 in non-video wards. For male knowledge and actions outcomes, we analysed 3051 spouses of pregnant women in video wards and 3880 in non-video wards. Tables S1, S2 and S3 in supplementary file 1 show details of the participant flow of women, children and men in the trial.

### Characteristics of participants

Tables S4, S5 and S6 in supplementary file 2 show the socio-demographic characteristics of women, children and men in video and non-video wards. As shown in the tables and highlighted in the footnotes to Tables S4, S5 and S6, there were some statistically significant differences between video and non-video wards. Women in the video wards were four times more likely to be from remote communities. Children in video wards were more likely to be from remote communities (five times), to have adolescent mothers (1.5 times) and to have mothers (1.5 times) and fathers (1.8 times) with some education. Male spouses of pregnant women in the video wards were more likely to be from remote communities (1.3 times), more likely to be aged less than 30 years (1.6 times) and less likely (0.7 times) to have a better income occupation.

### Outcomes and estimation of impact

#### Maternal outcomes

There were few differences in maternal outcomes between video and non-video wards (Table 1). Pregnant women from video wards were significantly more likely to have discussed pregnancy and childbirth often with their husbands, compared with women from non-video wards. This association remained significant in a multivariate model including the socio-economic variables shown in Table S4 in supplementary file 2. Women in video wards were also more likely not to experience verbal abuse during their pregnancy, but this difference was not significant at the 5% level in a multivariate model including the socio-economic variables in Table S4.

**Table 1.** Maternal outcomes among women in video wards and non-video wards.

Outcomes	Fraction (%)		OR (95% CIca)	
	Video wards	Non-video wards	Bivariate^a^	Adjusted^b^
**Number of women giving birth**	3785	3899		
***Risk factors covered in the videos***				
Know at least 3 danger signs in pregnancy	53.4 (2020/3785)	68.1 (2657/3899)	0.53 (0.23–1.24)	0.58 (0.23–1.50)
Reduced heavy work before 3^rd^ trimester	58.2 (2157/3706)	47.9 (1837/3834)	1.51 (0.79–2.89)	1.41 (0.61–3.26)
Often discussed pregnancy and birth with spouse	48.1 (1816/3772)	28.6 (1107/3871)	**2.32** (**1.52–3.54)**	**2.22** (**1.07–4.59)**
No verbal abuse during pregnancy	84.7 (3190/3768)	62.4 (2428/3888)	**3.32** (**1.19–9.24)**	3.27 (0.93–11.48)
No physical violence in pregnancy	96.6 (3632/3760)	96.3 (3734/3879)	1.10 (0.60–2.01)	1.24 (0.62–2.46)
***Pregnancy***				
Four or more antenatal care visits	55.4 (2039/3678)	51.1 (1969/3853)	1.19 (0.51–2.79)	1.27 (0.63–2.55)
No persistent headache	84.9 (3215/3785)	71.4 (2785/3899)	2.26 (0.70–7.26)	2.49 (0.64–9.59)
No swelling hands, feet, face	92.2 (3488/3785)	86.6 (3378/3899)	1.81 (0.62–5.28)	1.97 (0.61–6.42)
No vaginal bleeding	95.8 (3627/3785)	94.7 (3691/3899)	1.29 (0.63–2.64)	1.36 (0.67–2.75)
***Childbirth***				
Childbirth in health facility	51.9 (1758/3386)	56.2 (1942/3454)	0.84 (0.20–3.50)	0.92 (0.32–2.61)
No sepsis after birth	52.7 (2748/3771)	47.3 (2486/3880)	1.54 (0.95–2.50)	1.74 (0.83–3.65)

OR = odds ratio; 95% CIca = 95% confidence interval, adjusted for clustering by ward.

**Bold font** indicates an association significant at the 5% level.

^a^ Association from bivariate analysis of the outcome and the intervention (video vs. non-video).

^b^ Adjusted association from a multivariate model including the socio-economic characteristics shown in Table S4 in supplementary file 2: remote community vs. rural or urban; household food sufficiency in last week; household head with some formal education; woman with some formal education.

#### Child outcomes

There were few significant differences in child outcomes between video and non-video wards (Table 2). Mothers of children in the video wards were more likely to say they would not give anti-diarrhoeal medicine to a child, and children with diarrhoea in video wards were less likely to be given anti-diarrhoeal medicine. But these associations were not statistically significant in multivariate models including the socio-economic variables in Table S5 in supplementary file 2.

**Table 2.** Outcomes for children aged 12–18 months born to mothers in video and non-video wards.

Outcomes	Fraction (%)		OR (95% CIca)	
	Video wards	Non-video wards	Bivariate^a^	Adjusted^b^
**Diarrhoea prevention**				
*Knowledge and behaviour for prevention*				
Mother mentions poor hygiene as cause of diarrhoea	56.6 (611/1080)	61.1 (420/687)	0.83 (0.46–1.48)	1.19 (0.53–2.66)
Household has better hygiene (no garbage, sewage or excreta observed)	65.8 (546/830)	67.7 (354/523)	0.92 (0.39–2.15)	0.74 (0.29–1.92)
Household with clean, covered & raised drinking water container observed	48.3 (401/830)	65.6 (343/523)	0.49 (0.17–1.45)	0.44 (0.18–1.10)
Household treats drinking water	38.7 (319/825)	32.8 (171/522)	1.29 (0.54–3.10)	1.21 (0.56–2.62)
*Diarrhoea prevalence*				
Child had diarrhoea in last 15 days	17.8 (170/953)	16.4 (100/609)	1.11 (0.67–1.82)	0.95 (0.58–1.55)
**Diarrhoea management**				
*Knowledge and behaviour*				
Mother thinks child with diarrhoea should be given more fluid & continued feeding	44.6 (482/1080)	31.7 (218/687)	1.73 (0.72–4.15)	1.64 (0.84–3.18)
Mother would not give a child medicine to stop diarrhoea	47.1 (509/1080)	20.1 (138/687)	**3.55** (**1.61–7.80)**	4.52 (0.73–28.00)
*Management of last episode of diarrhoea*				
Given more fluids and continued feeding during last episode of diarrhoea	36.5 (62/170)	56 (56/100)	0.45 (0.10–2.13)	0.42 (0.15–1.19)
Not given any medicine to stop diarrhoea during last episode of diarrhoea	61.8 (105/170)	27 (27/100)	**4.37** (**1.63–11.73)**	2.63 (0.47–14.76)
**Childhood Immunisations**				
*Knowledge and behaviour of mother*				
Mother thinks it is worthwhile to immunise children	96.7 (1042/1078)	97.8 (670/685)	0.65 (0.22–1.93)	0.72 (0.34–1.52)
Mother discusses immunisation with spouse and family	94.7 (1023/1080)	90.4 (621/687)	1.91 (0.74–4.92)	2.02 (0.58–7.08)
Mother involved in decision about immunising the child	23.3 (253/1087)	30.8 (216/702)	0.68 (0.29–1.62)	0.65 (0.21–2.05)
*Immunisation status of child*				
Fully immunised	51.8 (564/1089)	46.7 (330/706)	1.22 (0.39–3.81)	1.60 (0.42–6.09)

OR = odds ratio; 95% CIca = 95% confidence interval, adjusted for clustering by ward.

**Bold font** indicates an association significant at the 5% level.

^a^ Association from bivariate analysis of the outcome and the intervention (video vs. non-video).

^b^ Adjusted association from a multivariate model including the socio-economic characteristics shown in Table S5 in supplementary file 2: remote community vs. rural or urban; with adolescent mother; mother with any formal education; father with any formal education; mother with enough food in last week.

#### Outcomes among spouses of pregnant women

There were few differences in knowledge and attitudes of male spouses between video wards and non-video wards (Table 3). Male spouses in video wards were significantly more likely to know to give more fluids and continued feeding to a child with diarrhoea. This difference remained significant in a multivariate model including the socio-economic variables shown in Table S6 in supplementary file 2.

**Table 3.** Knowledge and attitudes among male spouses in video wards and non-video wards.

Outcomes	Fraction (%)		OR (95% CIca)	
	Video wards	Non-video wards	Bivariate^a^	Adjusted^b^
***Related to maternal health***				
Know 3 danger signs in pregnancy	39 (1190/3051)	42.3 (1643/3880)	0.87 (0.33–2.29)	0.91 (0.38–2.18)
Think women should reduce heavy work before 3^rd^ trimester	81.3 (2453/3017)	78.7 (3054/3880)	1.18 (0.62–2.23)	1.19 (0.58–2.43)
Often discussed pregnancy and childbirth with spouse	81.3 (2454/3017)	92.6 (3593/3880)	0.35 (0.06–1.94)	0.38 (0.05–2.77)
***Related to child health***				
Mention poor hygiene as cause of childhood diarrhoea	76.9 (2023/2630)	75.0 (2711/3636)	1.11 (0.76–1.63)	1.12 (0.78–1.61)
Know to give child with diarrhoea more fluids and continued feeding	56.8 (1495/2630)	45.7 (1654/3616)	**1.56** (**1.14–2.14)**	**1.61** (**1.21–2.13)**
Would not give child with diarrhoea medicine to stop the diarrhoea	23.3 (614/2630)	21.5 (779/3616)	1.11 (0.53–2.34)	0.96 (0.50–1.85)
Think it is worthwhile to immunise children	97.5 (2563/2630)	98.5 (3563/3616)	0.57 (0.21–1.56)	0.52 (0.22–1.23)

OR = odds ratio; 95% CIca = 95% confidence interval, adjusted for clustering by ward.

**Bold font** indicates an association significant at the 5% level.

^a^ Association from bivariate analysis of the outcome and the intervention (video vs. non-video).

^b^ Adjusted association from a multivariate model including the socio-economic characteristics shown in Table S6 in supplementary file 2: remote community vs. rural or urban; household with enough food in the last week; with some formal education; with a better income occupation; aged 30 years or less.

### Qualitative appreciation of the videos

Female home visitors who conducted visits using videos thought they helped pregnant women to understand about the risk factors. They said the Hausa soap opera style meant people enjoyed and could relate to the videos.

The videos help the pregnant women understand the risk factors and take action more than if I had just explained. People don’t forget easily what they see. (Female home visitor)

Most of the women were excited watching the videos because they entertained them. While they watched, they laughed, nodded their heads sometimes in agreement with a statement, other times when sympathizing with a scene. (Female home visitor)

Male home visitors who used videos in their visits felt that the videos helped to increase men's awareness on maternal risk factors, although sometimes the men needed additional explanation.

Yes! [Using the videos] helps a lot in educating men and they fully understand the content of the videos. (Male home visitor)

All the 12 women and men from urban, rural and remote communities who viewed the videos ‘cold’ apparently understood what they showed. They were able to explain some key contents.

Women who continue doing heavy work during pregnancy are at risk. They might give birth to small and weak babies or give birth before time. (A female viewer, after watching the video on heavy work during pregnancy)

She (a character in the video) explained the mixture of salt and sugar to be added in the water as a solution for diarrhea treatment. (A male viewer after watching the video on childhood diarrhoea)

## Discussion

The video edutainment in the home visits was well received by households but was generally not associated with improved outcomes compared with visits without the videos. Just two outcomes were significantly better in video wards: discussion between spouses on pregnancy and childbirth, and male knowledge about home management of childhood diarrhoea. The home visitors valued the videos and felt they helped sustain people's interest and increased their understanding about the risk factors. However, these home visitors did not visit homes in non-video wards, so did not have direct experience of the understanding of households who did not view the videos.

Several studies have reported an impact of video edutainment in home visits on knowledge and behaviours. A controlled trial of a home visits intervention among Mexican-origin families in the USA reported improved outcomes of maternal diet and behaviours.^7^ The intervention used a combination of videos, discussions and workbooks to engage families; experienced health workers visited each family 11 times and placed 4 phone calls to them. The delayed-intervention control group did not receive any home visits. In Thailand home visits including edutainment videos were associated with improvements in knowledge and perceptions of mothers about childhood immunisations.^8^ The study used a before-after design; the comparison group were the same households prior to the intervention.

Our study differs from these two studies in that households in our control group had visits with the same content and discussion as the intervention group, rather than having no visits. We measured the *added value*, if any, of the edutainment video over and above the value of the visit. As previously reported, the home visits, with and without videos, led to significant improvements in maternal and child health outcomes and in male knowledge and actions.^13–15^

Studies of the use of videos in community interventions report that videos work well when people can identify with characters and story lines. In a community-based behaviour change study on maternal and newborn health in Ethiopia, the local project team contributed to scripting and production of videos.^5^ Researchers recruited local residents and actors for the videos and piloted the videos in communities. People who watched the videos said that the videos, based on typical local characters, took them into familiar situations they could easily relate to. People who attended showings of the videos reported more favourable attitudes and behaviour around maternal and newborn health.^5^ A qualitative study in Uganda found that culture and context specific videos were effective in communicating information on maternal, newborn and child health in rural communities.^4^ The videos in our home visits were locally scripted; feedback from the home visitors suggested this made them interesting and convincing for household viewers.

The home visits took place in the context of a research study, with intensive field training, and strict monitoring and supervision of home visitors. The careful training of home visitors in *non-video* wards helped them engage households in a comprehensive discussion about the maternal and child health risk factors addressed in the visits. In a wider rollout of the home visits in a service context such intensive training and support for home visitors will probably not be available. In this context the video edutainment docudramas might be useful and add value. The positive view of the home visitors about the utility of the videos is encouraging, suggesting that they would be willing to use them in a wider rollout of the home visits program. Future research could examine the impact of the video edutainment in a more routine service context, with less time for detailed verbal explanation and discussion from home visitors.

### Strengths and limitations

The study design, with random allocation of video edutainment to half the visited wards, allowed us to test specifically the added value of the video edutainment element of the home visits. Other studies of video edutainment as part of home visits have not attempted to do this. Although the trial randomly allocated wards to receive videos in the home visits or not, there were some significant socio-economic differences between video and non-video wards. We took account of these differences in a multivariate analysis which included these factors, and they did not explain the lack of effect of video exposure. But there may have been other unmeasured differences between the video and non-video wards among the small number of wards randomised. Higher exposure to the videos in repeated visits might have increased their impact but we were not able to test this possibility. We did not collect information directly from households about their experience and views of the videos; this would be a useful focus for future research.

## Conclusion

The home visits, with and without video edutainment, improved maternal and child health outcomes and male knowledge and attitudes. Video edutainment added to home visits implemented in the context of a research trial did not increase the measured impact of the visits. However, the videos may be a useful component of home visits when scaled up as a routine service with limited field support and training available to home visitors.

## Supplemental Material

sj-docx-1-dhj-10.1177_20552076241228408 - Supplemental material for Added value of video edutainment on android handsets in home visits to improve maternal and child health in Bauchi State, Nigeria: Secondary analysis from a cluster randomised controlled trial Click here for additional data file. Supplemental material, sj-docx-1-dhj-10.1177_20552076241228408 for Added value of video edutainment on android handsets in home visits to improve maternal and child health in Bauchi State, Nigeria: Secondary analysis from a cluster randomised controlled trial by Umaira Ansari, Khalid Omer, Amar Aziz, Yagana Gidado, Hadiza Mudi, Ibrahim Sabo Jamaare, Neil Andersson and Anne Cockcroft in DIGITAL HEALTH

sj-docx-2-dhj-10.1177_20552076241228408 - Supplemental material for Added value of video edutainment on android handsets in home visits to improve maternal and child health in Bauchi State, Nigeria: Secondary analysis from a cluster randomised controlled trial Click here for additional data file. Supplemental material, sj-docx-2-dhj-10.1177_20552076241228408 for Added value of video edutainment on android handsets in home visits to improve maternal and child health in Bauchi State, Nigeria: Secondary analysis from a cluster randomised controlled trial by Umaira Ansari, Khalid Omer, Amar Aziz, Yagana Gidado, Hadiza Mudi, Ibrahim Sabo Jamaare, Neil Andersson and Anne Cockcroft in DIGITAL HEALTH
