# Supplementary material for: Added value of video edutainment on android handsets in home visits to improve maternal and child health in Bauchi State, Nigeria: Secondary analysis from a cluster randomised controlled trial
Source: Digit Health. 2024 Feb 13;10:20552076241228408. doi: 10.1177/20552076241228408 (PMC10865940; doi:10.1177/20552076241228408)
Supplement: sj-docx-1-dhj-10.1177_20552076241228408 - Supplemental material for Added value of video edutainment on android handsets in home visits to improve maternal and child health in Bauchi State, Nigeria: Secondary analysis from a cluster randomised controlled trial [file sj-docx-1-dhj-10.1177_20552076241228408.docx]

**Supplementary File 1**

Table S1. Trial flow of pregnant women

|  | **Wave 1** | **Wave 2** | **Wave 3** | **Total** |
| --- | --- | --- | --- | --- |
| ***Video wards*** |  |  |  |  |
| Visited at least once | 2332 | 1793 | 4537 | 8662 |
| Not due for delivery by the cut-off point^1^ | 740 | 660 | 2464 | 3864 |
| Not due for a follow-up visit ^2^ | - | - | 123 | 123 |
| Lost to follow-up | 290 | 247 | 351 | 888 |
| Maternal deaths | 0 | 2 | 0 | 2 |
| **Number for analysis** | 1302 | 884 | 1599 | 3785 |
|  |  |  |  |  |
| ***Non-video wards*** |  |  |  |  |
| Visited at least once | 1513 | 1528 | 5685 | 8726 |
| Not due for delivery by the cut-off point^1^ | 511 | 568 | 2541 | 3620 |
| Not due for a follow-up visit^2^ | - | - | 68 | 68 |
| Lost to follow-up | 443 | 405 | 286 | 1134 |
| Maternal deaths | 1 | 2 | 2 | 5 |
| **Number for analysis** | 558 | 553 | 2788 | 3899 |

1 The cut-off point was one year after the intervention started.

2 In wave 3, some pregnant women were not due for a follow-up visit before the trial ended.

Table S2. Trial flow of children (12-18 months old)

|  | **Wave 1** | **Wave 2** | **Wave 3** | **Total** |
| --- | --- | --- | --- | --- |
| ***Video wards*** |  |  |  |  |
| Live births | 1197 | 843 | 1533 | 3573 |
| Children reported dead | 0 | 7 | 2 | 9 |
| Not due for follow-up | 0 | 0 | 147 | 147 |
| Not eligible for age | 0 | 0 | 1308 | 1308 |
| Lost to follow-up | 553 | 465 | 7 | 1025 |
| **Number for analysis** | 644 | 371 | 75 | 1090 |
|  |  |  |  |  |
| ***Non-video wards*** |  |  |  |  |
| Live births | 662 | 522 | 2485 | 3669 |
| Children reported dead | 2 | 10 | 4 | 16 |
| Not due for follow-up | 0 | 0 | 155 | 155 |
| Not eligible for age | 0 | 0 | 2161 | 2161 |
| Lost to follow-up | 263 | 353 | 15 | 631 |
| **Number for analysis** | 397 | 159 | 150 | 706 |

Table S3. Trial flow of spouses of pregnant women (visited at least twice)

|  | **Wave 1** | **Wave 2** | **Wave 3** | **Total** |
| --- | --- | --- | --- | --- |
| ***Video wards*** |  |  |  |  |
| Visited at least once | 1507 | 1410 | 3016 | 5933 |
| Not due for follow-up^1^ | 0 | 0 | 1561 | 1561 |
| Lost to follow-up after initial visit | 613 | 398 | 310 | 1321 |
| **Number for analysis** | 894 | 1012 | 1145 | 3051 |
|  |  |  |  |  |
| ***Non-video wards*** |  |  |  |  |
| Visited at least once | 1216 | 1200 | 3127 | 5543 |
| Not due for follow-up^1^ | 0 | 0 | 607 | 607 |
| Lost to follow-up after initial visit | 439 | 324 | 293 | 1056 |
| **Number for analysis** | 777 | 876 | 2227 | 3880 |

1 In wave 3, some men were not due for a follow-up visit before the trial ended.
