# Supplementary material for: Added value of video edutainment on android handsets in home visits to improve maternal and child health in Bauchi State, Nigeria: Secondary analysis from a cluster randomised controlled trial
Source: Digit Health. 2024 Feb 13;10:20552076241228408. doi: 10.1177/20552076241228408 (PMC10865940; doi:10.1177/20552076241228408)
Supplement: sj-docx-2-dhj-10.1177_20552076241228408 - Supplemental material for Added value of video edutainment on android handsets in home visits to improve maternal and child health in Bauchi State, Nigeria: Secondary analysis from a cluster randomised controlled trial [file sj-docx-2-dhj-10.1177_20552076241228408.docx]

**Supplementary File 2**

Table S4. Characteristics of women in video wards and non-video wards

| **Characteristic** | **Percent (fraction) of the women** | | **OR (95% CIca)** |
| --- | --- | --- | --- |
|  | **Video wards** | **Non-video wards** |  |
| Number of women | 3785 | 3899 |  |
| From remote community* | 18.7  (706/3785) | 5.2  (202/3899) | **4.20 (3.60-4.89)** |
| Household with enough food in last week | 96.6  (3542/3710) | 97.2  (3713/3821) | 0.83 (0.64-1.07) |
| Household head with some formal education | 59.2  (2144/3619) | 61.2  (2290/3742) | 0.92 (0.84-1.01) |
| Woman with some formal education | 55.0  (2079/3783) | 54.4  (2116/3893) | 1.02 (0.94-1.12) |

OR= odds ratio; 95% CIca = cluster-adjusted 95% confidence interval.

**Bold font** indicates a difference significant at the 5% level

*Women in video wards were significantly more likely to be from a remote community (vs rural or urban community)

Table S5. Characteristics of children aged 12-18 months born to mothers in video wards and non-video wards

| **Characteristic** | **Percent (fraction) of the children** | | **OR (95% CIca)** |
| --- | --- | --- | --- |
|  | **Video wards** | **Non-video wards** |  |
| Number of children | 1090 | 706 |  |
| From remote community* | 31.1  (339/1090) | 7.8  (55/706) | **5.34 (4.03-7.08)** |
| With adolescent mother (14-19 years)* | 16.4  (179/1090) | 11.5  (81/706) | **1.52 (1.15-2.01)** |
| Mother with some formal education* | 42.7  (465/1088) | 33.6  (234/696) | **1.47 (1.21-1.80)** |
| Father with some formal education* | 51.8  (563/1086) | 37.4  (257/688) | **1.81 (1.49-2.19)** |
| Mother with enough food in last week | 96.4  (1029/1067) | 96.9  (593/612) | 0.87 (0.50-1.52) |

OR= odds ratio; 95% CIca = cluster-adjusted 95% confidence interval.

**Bold font** indicates a difference significant at the 5% level

*Children in the video wards were significantly more likely: to be from a remote community (vs rural or urban community); to have an adolescent mother; to have a mother with some formal education; and to have a father with some formal education

Table S6. Characteristics of male spouses of pregnant women in video wards and non-video wards

| **Characteristic** | **Percent (fraction) of the spouses** | | **OR (95% CIca)** |
| --- | --- | --- | --- |
|  | **Video wards** | **Non-video wards** |  |
| Number of male spouses | 3051 | 3880 |  |
| From remote community* | 18.5  (565/3051) | 14.6  (566/3880) | **1.33 (1.17-1.51)** |
| Household with enough food in last week | 96.6  (2824/2924) | 95.8  (3600/3757) | 1.23 (0.9 - 1.59) |
| Male spouse with some formal education | 73.3  (2151/2934) | 75.5  (2641/3497) | 0.89 (0.80-1.00) |
| Male spouse with better income occupation* | 47.6  (1398/2935) | 55.2  (1931/3500) | **0.74 (0.67-0.82)** |
| Male spouse aged 30 years or less* | 24.6  (721/2936) | 17  (598/3510) | **1.59 (1.40-1.79)** |

OR= odds ratio; 95% CIca = cluster-adjusted 95% confidence interval.

**Bold font** indicates a difference significant at the 5% level

*Men (male spouses) in the video wards were significantly more likely: to be from a remote community (vs rural or urban); to be aged 30 years or less. They were significantly less likely to have a better income occupation.
